# Development of ClickClinica: a novel smartphone application to generate real-time global disease surveillance and clinical practice data

**DOI:** 10.1186/1472-6947-13-70

**Published:** 2013-07-02

**Authors:** Benedict Daniel Michael, David Geleta

**Affiliations:** 1 Institute of Infection and Global Health, University of Liverpool, 1st Floor, Ronald Ross Building, 8 West Derby Street, Liverpool L69 7BE, UK; 2 Department of Computer Science, University of Liverpool, Ashton Building, Ashton Street, Liverpool L69 3BX, UK

**Keywords:** Epidemiology, Audit, Clinical practice, Quality improvement, iPhone, Smart phone, Application, Guidelines

## Abstract

**Background:**

Identification and tracking of important communicable diseases is pivotal to our understanding of the geographical distribution of disease, the emergence and spread of novel and resistant infections, and are of particular importance for public health policy planning. Moreover, understanding of current clinical practice norms is essential to audit clinical care, identify areas of concern, and develop interventions to improve care quality.

However, there are several barriers to obtaining these research data. For example current disease surveillance mechanisms make it difficult for the busy doctor to know which diseases to notify, to whom and how, and are also time consuming. Consequently, many cases go un-notified. In addition assessments of current clinical practice are typically limited to small retrospective audits in individual hospitals.

Therefore, we developed a free smartphone application to try to increase the identification of major infectious diseases and other acute medical presentations and improve our understanding of clinical practice.

**Description:**

Within the first month there were over 1000 downloads and over 600 specific disease notifications, coming from a broad range of specialities, grades and from all across the globe, including some resource poor settings.

Notifications have already provided important information, such as new cases of TB meningitis, resistant HIV and rabies, and important clinical information, such as where patient with myocardial infarctions are and are not receiving potentially life-saving therapy.

The database generated can also answer new, dynamic and targeted questions. When a new guideline is released, for example for a new pandemic infection, we can track, in real-time, the global usage of the guideline and whether the recommendations are being followed. In addition this allows identification of where cases with key markers of severe disease are occurring. This is a potential resource for guideline-producing bodies, clinical governance and public health institutions and also for patient recruitment into ongoing studies.

**Conclusions:**

Further parallel studies are needed to assess the clinical and epidemiological utility of novel disease surveillance applications, such as this, with direct comparisons made to data collected through routine surveillance routes.

Nevertheless, current disease surveillance mechanisms do not always comprehensively and accurately reflect disease distribution for many conditions. Smartphone applications, such as ClickClinica, are a novel approach with the potential to generate real-time disease surveillance data that may augment current methods.

## Background

Improved understanding of the development and distribution of novel and resistant infections requires robust and readily utilised identification and tracking mechanisms. This is fundamental for adequate public health policy planning [1]. In addition, a clear understanding of current clinical practice norms is necessary to audit clinical care, identify areas of concern and develop interventions to improve care quality [2].

Whilst many countries have national disease surveillance programmes for major communicable diseases, or ‘notifiable diseases’, the work of our group and others has identified several areas of sub-optimal notification, even in the developed countries. For example, it is estimated by the Health Protection Agency (HPA), that for each laboratory confirmed cases of the major gastrointestinal infection, norovirus, there are up to a further 288 unreported cases [3]. Furthermore, a UK-wide prospective longitudinal study found that, for gastrointestinal infection overall, for every one case notified to national surveillance, there were 10 GP consultations and 147 community cases [4].

With regards to the major neurological infection, there were only 18 cases of acute encephalitis notified to the HPA in 2007 [5]. However, during a 3 month study conducted in the same year in only 10 adult hospitals we identified 13 cases of encephalitis [6]. In addition, the notification of acute viral meningitis between 2004–2006 was approximately 1381 per year [5]. During this same period in a single 950 bed general non-specialist NHS teaching hospital, we identified 68 hospitalised patients with viral meningitis [7]. If extrapolated across the 159,386 NHS beds over this same time period, the estimated number of cases would be nearly three times larger (n = 3,802 cases). [8] Indeed several studies have reported that the true incidence of meningitis in the UK may be 10–14 times higher than notified figures [9-11].

Nevertheless, microbiological notification of specific organisms which have been identified maybe somewhat better, as there are clear notification standard operating procedures in most laboratories. However, this may falsely skew epidemiological data to favour particular organisms of interest. For example, in 2011, 263 (48.8%) of all acute meningitis notifications to the HPA were due to *Neisseria meningitidis*[5]. Whereas, in disease-specific, rather than organism-specific, surveillance programmes in developed countries only around 13% of all meningitis cases are due to *Neisseria meningitidis*[12]. Furthermore, overall bacteria may only account for up to 26% of meningitis [13].

The Centre for Disease Control and Prevention (CDC) has brought together several reporting systems, including for HIV and TB, into a single place through the National Electronic Disease Surveillance System (NEDSS) [14]. However, this approach still requires each individual to upload their data and does not collect data automatically from doctors on the front line of patient care.

Suboptimal notification from doctors on the front line may potentially be due to lack of awareness that the condition is a notifiable one, lack of knowledge of the procedure for notification and/or limited resources to facilitate the notification process, such as limited time. From previous work we have found that simple interventions, such as providing the appropriate bottle for virus identification or for cancer cell identification within the kits doctors use when performing a lumbar puncture, we could increase the identification of viruses and cancer cells [15,16]. Although most doctors knew the importance of sending these types of sample, making it less time-consuming to collect them resulted in real changes in practice and disease identification.

In addition assessments of current clinical practice are typically limited to small retrospective audits of case notes in individual hospitals. Therefore, they only provide delayed information, which is specific to a locality rather than reflecting wider healthcare practice [17].

To start to address some of these issues and to enhance disease surveillance, novel technological approaches, such as ‘crowdsourcing’ are being increasingly used. For example, two recent papers report attempts to use Google © trends data to assess influenza incidence and compared this with CDC data. One identified good correlation between Google © influenza search trends and CDC notifications and one utilised these data to develop a mathematical model to predict influenza outbreaks 7 weeks prior to the occurrence. [18,19] However, whilst this approach utilises large datasets, which are required to generate such models, the veracity of the data is less robust than that submitted by clinicians.

Medical technology, such as ‘Apps’ for smartphones are revolutionising the way doctors practice medicine, increasing dissemination of research and making clinical guidelines more widely available [20]. However, the majority of these support only unidirectional traffic of information (e.g. simply dissemination of guidelines or monitoring of an individual’s health parameters, such as blood pressure recordings, and relaying this information to their individual doctor) [21]. A structured Medline search was conducted for papers between 1985-current in any language using the search terms: “smartphone” OR “phone” OR “application” WITH “disease surveillance” OR “epidemiology”. A further search was conducted using the above search terms in the Apple App Store ©. However, neither approach identified any applications, which allow for the bidirectional flow of data from a central database to clinicians (i.e. clinical guidelines) and from clinicians to a central database (i.e. disease notifications).

Therefore, we set out to develop a novel smartphone application with bidirectional information traffic to try to address both of these issues; firstly to improve disease surveillance though increasing the notification of major infectious and other acute diseases and secondly to improve our understanding of current clinical practice (https://sites.google.com/site/clickclinicarx/).

## Construction and content

The application brings together the clinical guidelines from across guidelines groups in a single place and is made freely available for the iPhone. These include guidelines from large national and international bodies such as the National Institute for Health and Clinical Excellence (NICE), HPA, CDC, the Resuscitation Council, the World Health Organization (WHO) and many others, Depending on the size of each guideline, they are either stored in whole or as a clinical summary of the key points as a PDF. Therefore, the clinician does not require internet access at the time they wish to get the information. Where guidelines were given in summary format or further multimedia material is available, a hyperlink to the full online guideline is provided. In a similar way to our previous projects, in which we have found that interventions that reduce the time required from doctors have the greatest impact, the app does not require any additional effort from the doctor than simply reading the guidelines they want to read anyway [15,16]. Indeed, by making them readily available and clearly indexed, it is easier for the doctor to use the app to read a section of the guidelines than it is to perform an internet search, then find the website, then find the document, then download the PDF, then scroll through to find the page of interest.

**Figure 1:**
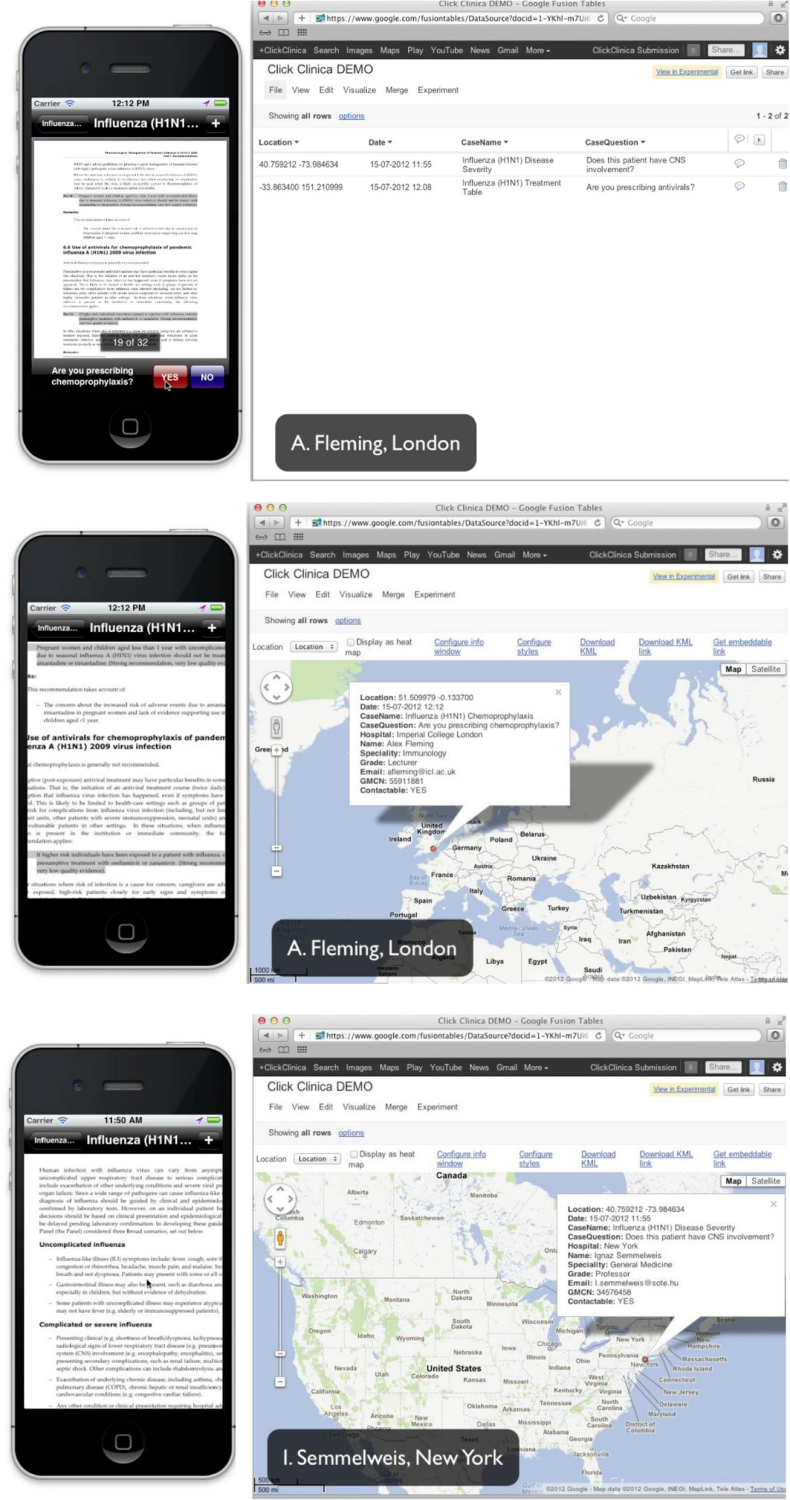
Guideline and database views, with questions being answered.

**Figure 2:**
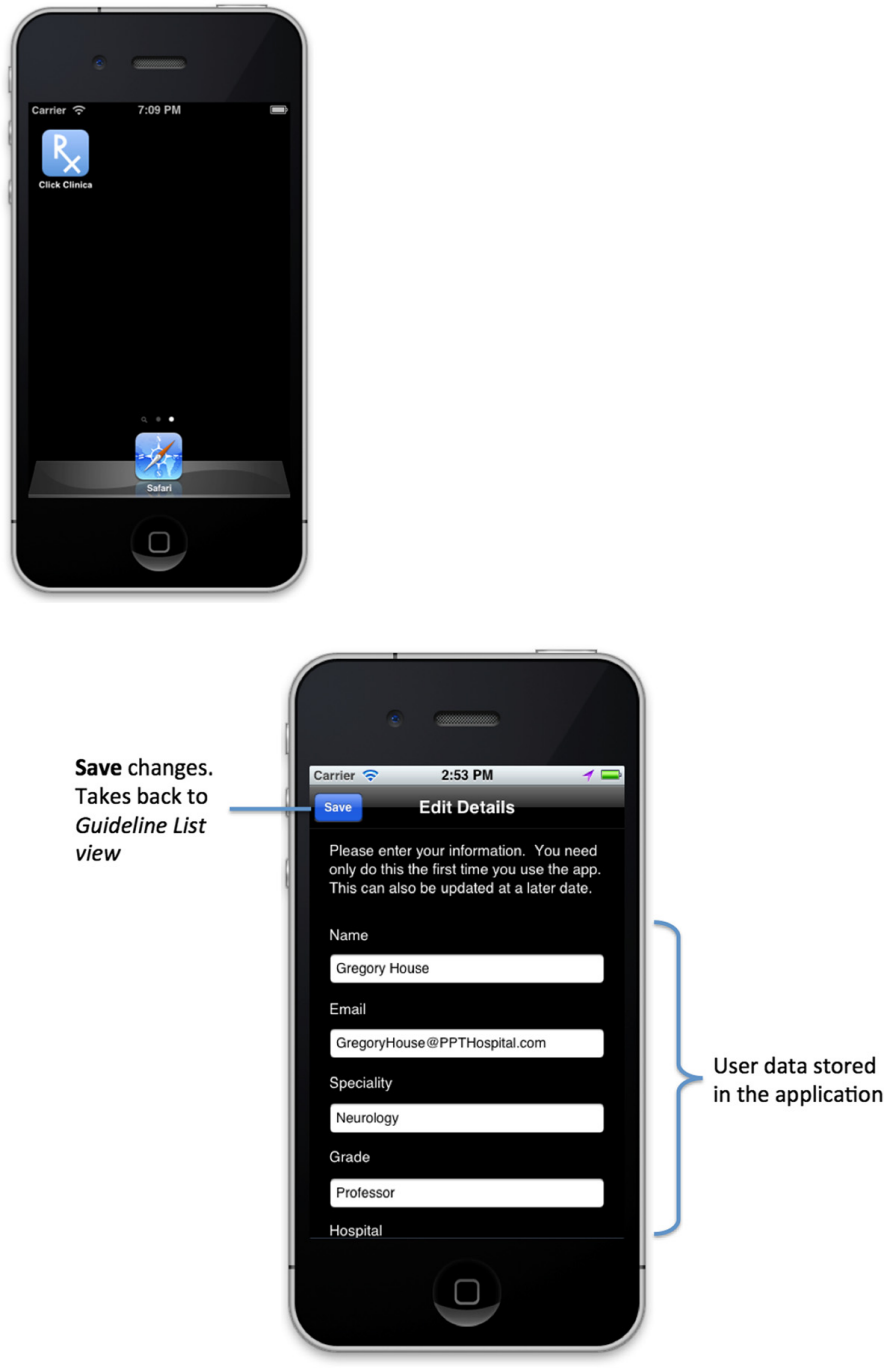
Launching the ClickClinica© application and initial registration.

In reading guidelines on the application, the app has the potential to generate a real-time global disease surveillance database based on the usage. Also from a brief question that appears on the bottom of the screen when viewing a specific disease, more detailed information on either important epidemiological or clinical care quality issues can be collected. One example is that when a doctor is viewing a guideline on H1N1 influenza, our central database is made aware that the guideline is being read and the doctor’s grade and hospital. Moreover, on clicking yes in the ‘question box’ at the bottom of the screen, such as “Are you prescribing chemoprophylaxis?” we are immediately also notified that the doctor has answered the question and their name, specialty, GPS location and, if allowed, email address, medical registration number and also whether the doctor is happy to be contacted for research purposes regarding this case. In answering the question this ‘question box’ disappears providing more space for the guideline to be viewed (Figure 1). The doctor only needs to input their personal information once at the point of downloading the application (Figure 2). Therefore, the application allows us to immediately track the distribution of disease, the spread of outbreaks and their management. The question at the bottom of the screen is specific for each disease, or sub-section within the disease guideline.

For example, for influenza:

● When reviewing the front page, the question is “are you seeing a patient with H1N1?”

● When reviewing the resistance page within the influenza document the question is “does this patient have resistant virus?”

● When reviewing the treatment table within the influenza document the question is “are you prescribing antivirals?”

Whilst these questions are currently set centrally by the ClickClinica team, as the project expands we are collaborating with other research institutions to use our platform for them to include research questions of interest to their particular field. The data collected regarding this is made freely available to them to access in real-time through a dedicated terminal, including questions submitted by users. When the app is used in a situation where a clinician does not have an internet connection (be that Wi-Fi or wired internet) the notification is stored within the application and subsequently submitted to the central database automatically when an internet connection is established.

By obtaining comprehensive data on the usage of the app, we are able to include guidelines that reflect usage in future updates. In addition the application contains a ‘Flag it up’ button, which allows users to suggest guidelines they would like to see in future updates of the application.

To encourage use of the application, we have included guidelines to all the major acute medical presentations that are covered in competing paid-for applications and handbooks. Also, we have made it possible for clinicians to print, email or bookmark the guidelines directly from their smartphone. This also helps to foster guideline dissemination and the creation of a personalised app. To encourage users to answer the research questions we have included internal ClickClinica © certificates that can be used as evidence of involvement in research for their portfolio of continuing professional development. Also at any time the user can view their personalised index of recorded cases.

## Utility and discussion

The database generated from the usage of the app can also answer new, dynamic and targeted questions. When a new guideline is released (e.g. for a new pandemic infection) this app has the potential to track, in real-time, the global uptake and usage of the guideline. We can also assess whether the recommendations in that guideline (e.g. vaccine delivery) are being followed and where cases with key markers of severe disease (e.g. neurological involvement) are occurring. This is a potential resource of clinical information not only for guideline-producing bodies but also for clinical governance and public health institutions.

At the time of writing, the app has only been live for just over 4 weeks and has already received over 1000 downloads and over 600 specific disease notifications. The app did not receive any external funding and did not undergo a marketing campaign. Potential users were made aware of the app through the Apple App Store, within the ‘free medical applications’ category. In an effort to reduce spurious notifications, the app was designed such that it is equally efficient for the user remove the ‘question box’ from the screen by choosing to not notify as it is if they notify. Of the notifications, 578 (96%) have included an email address and 405 (70%) of these notifications have included consent to be contacted to provide further information for research purposes on the case they have notified.

Data quality is assessed firstly by the veracity of the user’s details (e.g. the data is treated as the weakest possible evidence of a disease notification when the report only contains the user’s name). The data is treated as most robust when a notification contains the user’s name, official NHS email address and General Medical Council number, which are crosschecked with the database. Furthermore, downstream data quality assessment is undertaken through an automated process to exclude multiple disease notifications during a short period of time (e.g. if the same disease is notified repeatedly within 10 minutes or multiple diseases are notified within 5 minutes) as such situations are likely to reflect users simply reading the guidelines for their interest or initially to ‘test out’ the app, rather than in relation to a specific patient. Nevertheless, we have endeavored to minimise these types of inappropriate notifications, as it is equally efficient, from a user’s point of view, to not notify, as it is to notify.

Specific disease notifications have come from a broad range of clinical specialities, including General Practice (25%), Emergency Medicine (15%), General Medicine (14%), and Anaesthetics, Paediatrics, Surgery, Infectious Diseases, Psychiatry, Cardiology, and Neurology.

**Figure 3:**
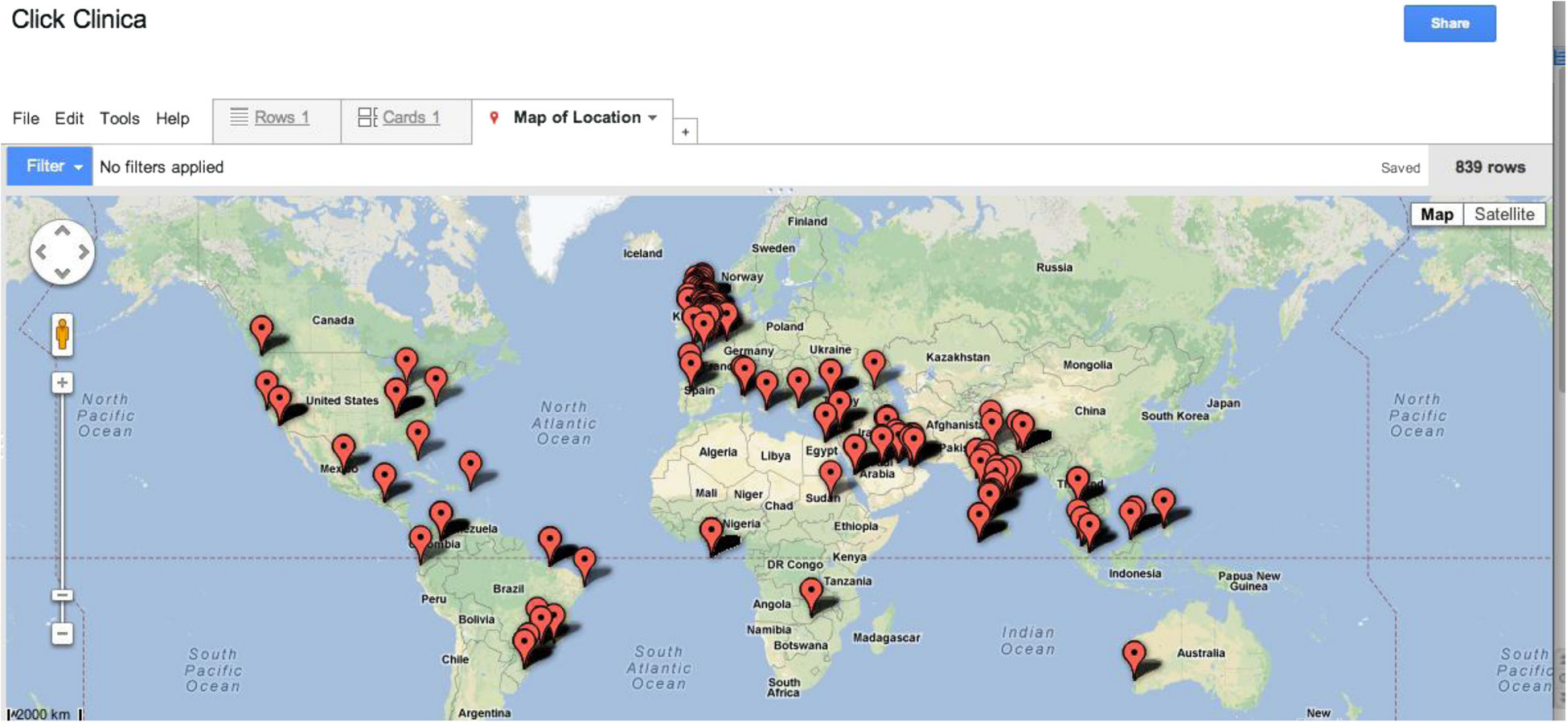
Real-time global disease database generation; Live 10.23 am, 5th November 2012.

Also, downloads and notifications have been coming from across the globe, including some resource-poor countries (Figure 3), and from a wide range of grades, including Foundation Doctors (24%), Medical Trainees (21%), Specialist Registrars (23%), Consultants (18%), and Professors/Lecturers (6%).

Our data collection approach allows us to collect both the denominator data (i.e. which guidelines are being read by whom) and the numerator data (i.e. specific disease notifications). Therefore, we are able to monitor usage of the app generally as well as usage of the app for disease notifications. This allows us to assess data collected as both simple frequencies and also as a proportion of usage for that individual user and also as a proportion of usage of the specific disease guideline of interest. Furthermore, these data can be visualised as a global or regional heat-map which can incorporate population data, for example disease notifications can be represented as an incidence within a population or even and incidence within a population exposed to a particular environmental risk factor. Notifications have already provided really important clinical information, for both disease surveillance and for clinical care quality assessment. Some examples of data collected are described in the table (Table 1).

**Table 1:** Examples of data collected for disease surveillance and clinical care quality assessment

**Notification type**	**Example(s)**
Disease Surveillance	Three new cases of TB meningitis: one in the UK and two in Pakistan
	Diagnosis of rabies in Myanmar
Clinical care quality	Of 48 patients with an ST-elevation myocardial infarction; seven received intravenous nitrates; only two received thrombolysis
	Of 21 patients with exacerbations of chronic obstructive pulmonary disease; five required non-invasive ventilation; only two were able to be managed in the community
	Of the eight patients with dementia, only three were prescribed an anticholinergic
	Of five patients with HIV, one required 2nd line therapy for drug-resistant disease
Assessing dissemination and education	23 doctors assessed a patient with suspected meningitis, decided whether to perform a lumbar puncture and then watched the video on how to do it

**Figure 4:**
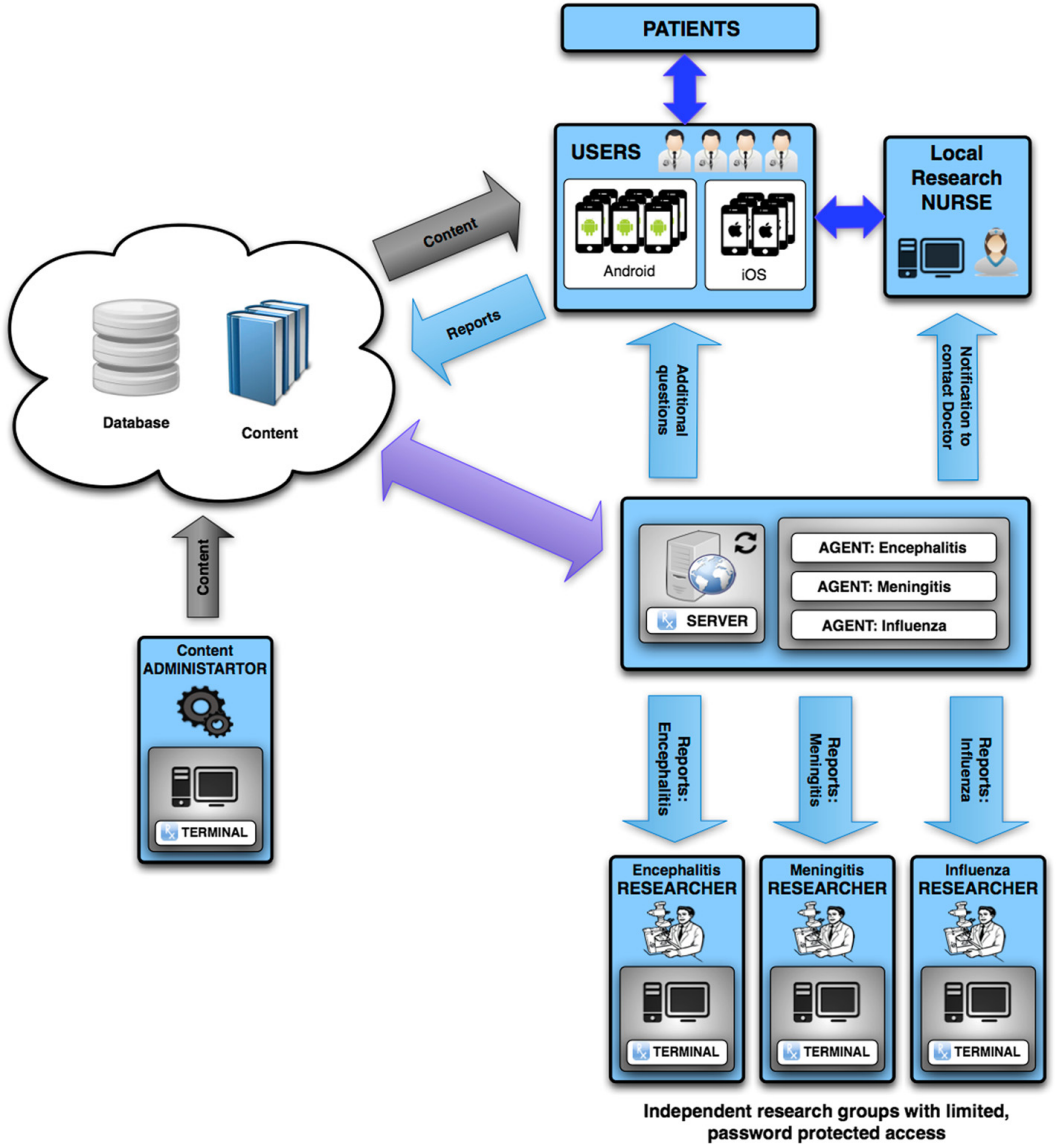
Schematic demonstrating data transfer of content and reports between users, researchers and the central team.

The application does not collect any patient-identifiable demographic data, but rather the data is of the doctor's practice, which they have agreed to provide at the point of installing the app. In addition, it also has the potential to increase recruitment into ongoing clinical studies. If a doctor is seeing a patient and reading a guideline for a disease in a hospital in which there is an ongoing study, then an automated system can generate an email to the doctor and the research nurse for that hospital allowing them to make contact to consent the patient to be recruited into that study (Figure 4). Therefore, no confidential or patient-identifiable information is made available centrally.

During this initial proof-of-concept pilot phase of the application, notifications from the individual user are available for them to review for themselves within the app itself. However, the wider database was only available to the central research team. Nevertheless, as there has been growing interest in the potential for this rich data source to inform future research, we have developed a number of research collaborations, including with researchers at the universities of Liverpool, San Diego and Oxford, through whom we are making the data relating to the conditions of interest readily accessible.

However, this approach to data collection is reliant on doctors using clinical guidelines. This will vary between disease areas in response to many factors, such as disease complexity or rarity, new guideline availability, individual clinician experience with the disease in question and available time. In addition, doctors do not typically refer to guidelines for every patient they see, particularly in areas where they are able to practice without seeking further information, and in these areas our data collection will be more limited. Nevertheless, clinicians regularly use guidelines and other reference texts to access information that is difficult to remember, such as drug dosages, which specific investigations are most sensitive and specific, or diseases that are not routinely seen. Furthermore, routine use of clinical guidelines, and indeed audit of their use, has an increasingly important role in day-to-day clinical practice. Moreover, as it does not require any additional time to notify a disease once they are reading the guideline, it is hoped that, if even only for specific disease areas, some useful data will be collected. Nevertheless, as novel technologies start to generate a new type of epidemiological data, new approaches to understanding and automating how best to interpret these data, including algorithm generation, will be required [22,23].

Future versions of the application will need to be developed for other smartphone platforms, such as the Google Android© market. As many doctors use smart phones on a daily basis there is at least the potential to access a wide cohort from whom to collect data. Moreover, future iterations could provide information for, and access data from, allied health care professionals, scientists and the wider public.

## Conclusions

It is unclear at this very early stage if this sort of approach can improve disease surveillance and clinical practice research, or indeed how many doctors would have to use such an app to create the critical mass to collected meaningful data. Further studies are needed to assess the data collected through these approaches in parallel with that collected through ongoing studies and routine surveillance approaches.

Nevertheless, current approaches appear to miss many cases and novel approaches, such as ClickClinica ©, may provide data to start to address this.

## Availability and requirements

The ClickClinica© database is held centrally and password encrypted. However, research, guideline-development and care quality groups can apply to access data for specific guidelines and diseases, and can also apply to include research questions or guidelines of interest to them in future ClickClinica © application updates.

## Competing interests

The author has no competing financial interests to disclose, the smartphone application, ClickClinica©, described is made freely available for download.

Following the submission of this manuscript, BDM has been awarded a research grant from the Institute of Infection and Global Health to utilise the app to assist with patient recruitment into studies of meningitis and gastrointestinal infection.

BDM is an NIHR Doctoral Research Fellow.

## Authors’ contributions

The application, ClickClinica © was conceived by BDM who also led the development of the application, including collating the guidelines, developing the research questions and analysing the data. The application was developed in collaboration with DG, who also had significant intellectual input in response to peer-review and the revision of this manuscript for publication and approved the final manuscript. Both authors read and approved the final manuscript.

## Pre-publication history

The pre-publication history for this paper can be accessed here:

http://www.biomedcentral.com/1472-6947/13/70/prepub
